# What Is a Hologenomic Adaptation? Emergent Individuality and Inter-Identity in Multispecies Systems

**DOI:** 10.3389/fpsyg.2020.00187

**Published:** 2020-03-04

**Authors:** Javier Suárez, Vanessa Triviño

**Affiliations:** ^1^LOGOS/BIAP, Department of Philosophy, University of Barcelona, Barcelona, Spain; ^2^Egenis, The Centre for the Study of Life Sciences, Department of Sociology, Philosophy and Anthropology, University of Exeter, Exeter, United Kingdom; ^3^Department of History of Science, Rey Juan Carlos University, Madrid, Spain

**Keywords:** holobiont, hologenome, microbiome, biological individuality, adaptation, emergence, inter-identity, metaphysics of biology

## Abstract

Contemporary biological research has suggested that some host–microbiome multispecies systems (referred to as “holobionts”) can in certain circumstances evolve as unique biological individual, thus being a unit of selection in evolution. If this is so, then it is arguably the case that some biological adaptations have evolved at the level of the multispecies system, what we call *hologenomic adaptations*. However, no research has yet been devoted to investigating their nature, or how these adaptations can be distinguished from adaptations at the species-level (genomic adaptations). In this paper, we cover this gap by investigating the nature of hologenomic adaptations. By drawing on the case of the evolution of sanguivory diet in vampire bats, we argue that a trait constitutes a hologenomic adaptation when its evolution can only be explained if the holobiont is considered the biological individual that manifests this adaptation, while the bacterial taxa that bear the trait are only opportunistic beneficiaries of it. We then use the philosophical notions of *emergence* and *inter-identity* to explain the nature of this form of individuality and argue why it is special of holobionts. Overall, our paper illustrates how the use of philosophical concepts can illuminate scientific discussions, in the trend of what has recently been called metaphysics of biology.

## Introduction

Adaptations are believed to be widespread in the biological world.^1^ The different types of beaks among bird species, the capacity of producing hemoglobin in vertebrates, or the ability to fly in some insects, are all considered adaptive traits. Yet, to properly characterize and recognize adaptations in nature is, notwithstanding, a difficult task for biologists due especially to the different ways this concept can be defined (Godfrey-Smith, 2001; Lloyd, 2017b). One of the most important issues that the concept of adaptation raises is that, since adaptations are usually considered adaptations *of an individual*, it is necessary to establish criteria to delineate the biological individuals that bear them before the identification of the adaptations becomes biologically feasible.

In this paper, we argue for a form of recognizing and explaining the evolution of some biological adaptations that result from the interaction between a multicellular host and its microbiome, whose discovery relies on the consideration of the holobiont as a biological individual. In particular, we appeal to the notions of *emergence* and *inter-identity* to shed light on the ancient debate about who is the individual for which adaptations evolve (Boogerd et al., 2005; Mitchell, 2012; Mossio et al., 2013; Moreno and Mossio, 2015; Triviño and Nuño de la Rosa, 2016; Canciani et al., 2019; Suárez and Triviño, 2019). We propose that the holobiont is the emergent individual that manifests the adaptations that underlie some specialized lifestyles (e.g., hematophagy, herbivory, etc.), an individual that we refer to as the *manifestor of adaptation* (Lloyd, 1992, 2001, 2017b). We further argue that the identity of the holobiont through time can only be established in terms of “inter-identity.” Importantly, we use the notions of “emergence” and “inter-identity” as the driving notions of our account, i.e., we use these concepts to illuminate some features of the individuality of holobionts that would be masked if the theoretical resources that they provide were ignored. By placing emphasis in these theoretical resources, our paper shows how biological evolution may occasionally give rise to forms of individuality (manifestors of adaptation) that go beyond the traditional boundaries of organisms.

In “Biological Individuals as Manifestors of Adaptation,” we motivate the necessity of developing an account of the holobiont as an individual that manifests biological adaptations and justify its relevance for biology. In “Sanguivory Diet in Vampire Bats,” we introduce a case study from biology about the evolution of sanguivory diet in bats of the species *Desmodus rotundus*. Drawing on some recent research by Mendoza et al. (2018), we show the existence of several hologenomic adaptations underlying the evolution of sanguivory, which suggests that the holobiont is the individual that manifests the adaptations and thus, the reason why the adaptations evolved in the first place. In “Holobionts as Emergent Individuals,” we argue for the emergent character of sanguivory, and the emergent character of the vampire bat holobiont. Relying on the philosophical notion of *emergence*, we argue that holobionts are emergent biological individuals, and explain the main metaphysical and biological implications of this conception of the holobionts. In “The Inter-Identity of the Holobiont,” we introduce the connection between individuality and identity and suggest an account of the temporal identity of the holobiont as a form of inter-identity that results from the causal-functional interaction between the host and its microbiome. Finally, we conclude by highlighting how our paper shows the ways in which metaphysics and biology can complement and help each other, in the fashion of what has been recently called *metaphysics of biology*.

## Biological Individuals as Manifestors of Adaptation

The debate concerning the characterization of *adaptation* is closely related to that of functions. Historically, this debate has been divided into two main positions: etiological and dispositional accounts (Millikan, 1989; Godfrey-Smith, 1993, 1994; Kitcher, 1993; Walsh, 1996; Mossio et al., 2009).^2^ According to *etiological* or *selected effects* accounts, a trait is an adaptation when its current presence in the organism is a consequence of some beneficial effect the trait performed in the past for the organisms in the particular lineage to which the organism belongs. In this sense, the presence of the trait can be explained in terms of its causal history, that is determined by the action of natural selection on the lineage where the trait originally appeared (Williams, 1966; Wright, 1973; Sober, 1984, 2000; Sober and Wilson, 2011). In this etiological sense of adaptation, it is assumed that the trait performed a function in the past on an organism and conferred the organism a fitness advantage. As a consequence, it was naturally selected on the lineage that the organism belongs to due to the fitness benefits it provided to its bearers, and that causal history is precisely what makes it to be an adaptation.

*Dispositional (or forward looking) accounts*, on the contrary, characterize adaptations as those traits of an organism that perform a function that contributes to a distinctive higher-level capacity of the organisms that bear them, irrespectively of their biological history (Mossio et al., 2009; see Moreno and Mossio, 2015, pp. 62–87, for a review on this topic). There are different forms of conceiving the higher-level systemic capacity, although it is generally assumed that fitness must be conceived as a propensity, whose goals are identified with survival and reproduction (Bigelow and Pargetter, 1987; Boorse, 2002). According to the dispositional account, therefore, a trait is an adaptive trait if it increases the fitness (survival and/or reproductive success) of the individuals that bear it (Bouchard, 2008, 2011; Triviño and Nuño de la Rosa, 2016). This definition of adaptation has also been called the “engineering notion,” in the sense that the traits will be considered adaptations if they make the individuals that bear them look as if they had a good engineering to fit their environment (Lloyd, 1992, 2001, 2017b).

Despite the differences between etiological and dispositional accounts, both definitions of adaptation assume the existence of a biological individual that either bears the adaptive trait *now* (dispositional account) or used to bear the adaptive trait *in the past* such that this is the reason why the trait exists now (etiological account). Following Elisabeth A. Lloyd, we will refer to the biological individual that bears the etiological trait as the *manifestor of adaptation* (Lloyd, 1992, 2001, 2017b).^3^ Recognizing the individual that manifests the adaptation is an essential task to properly identify the historical origins of the traits that are observed in the biological realm, and thus it is essential to distinguish the traits that are adaptive from those that are not.^4^

Generally, biological individuals have been equated to paradigmatic cases of multicellular organisms, such as mammals or birds. In these cases, identifying the individual that manifests the adaptation might be an easy task. For instance, it is easy to see that birds are the manifestors of the different types of beaks [think of the finches studied by Grant and Grant (1989, 2011)], or that each vertebrate manifests the ability to synthesize hemoglobin, for instance. In other cases, however, this task is not so easily performed. For instance, is the biological individual the polyp or the jellyfish? And what about the Portuguese man o’war? Is it an individual, or a colony of interdependent individuals? These cases seem more problematic, insofar as it is not clear how to delimit the boundaries of the organism, or what counts as an individual rather than many. These aspects substantially complicate the task of attributing adaptations (Pepper and Herron, 2008; Dupré and O’Malley, 2009; Clarke, 2010, 2013; Dupré, 2010, 2012; Wilson and Barker, 2013; Pradeu, 2016; DiFrisco, 2017; Lidgard and Nyhart, 2017).

In this regard, think for example of the barbed sting in honey bees. The sting seems to be a product of cumulative selection; that is, it is a structure that has evolved because natural selection has played a fundamental causal role in its evolution, i.e., natural selection is the reason why the trait is now in every honey bee. However, it seems at least perplexing to believe that the sting could be an adaptation of each honey bee, since its use can sometimes cause the death of its bearer. How is it possible that natural selection has caused the appearance of a structure whose use causes the death of its bearer? The initial perplexity, though, disappears when one considers the possibility that the individual that manifests the adaptation is not each honey bee, but the colony itself. To explain the evolution of structures such as barbed stings, some biologists appeal to the concept of *the superorganism* and multi-level selection (Okasha, 2006; Hölldobler and Wilson, 2009; Canciani et al., 2019). According to this approach, in some eusocial insect species such as honey bees, the colony is the manifestor of adaptation due to the complex cooperative organization. Because of this, traits that might be harmful to each individual insect can evolve because they are adaptive at the level of the colony, which would be the manifestor of adaptation in this particular case.

Symbiosis poses another interesting challenge for our conception of biological individuality (Brucker and Bordenstein, 2012, 2013a; Gilbert et al., 2012, 2017; Sapp, 2016; Stencel and Proszewska, 2017; Suárez, 2018, 2019). During the first two decades of the 21th century, discoveries concerning symbiotic relations given in different species such as coral reefs and their microbiome (Rosenberg et al., 2007; Thompson et al., 2015), or the *Nasonia* wasps and their microbiome (Brucker and Bordenstein, 2013b), together with the realization of the near omnipresence of symbiosis, have suggested the existence of new forms of individuality at the multispecies level (Bouchard, 2009, 2013, 2014). In this respect, the notion of “holobiont” has been recently coined to refer to the multispecies symbiotic assemblages composed by a host (animal or plant) plus its symbiotic microbiota. Under the umbrella of the so called *hologenome concept of evolution*, it has recently been argued that holobionts are biological individuals, a position that we will call the *individual view* (Zilber-Rosenberg and Rosenberg, 2008; Rosenberg and Zilber-Rosenberg, 2013, 2016; Bordenstein and Theis, 2016; Theis et al., 2016; Lloyd, 2017a; Roughgarden et al., 2017; Suárez, 2019).

The idea, notwithstanding, has been faced with some criticism on the basis that firstly, the hypothesis that the holobiont is a biological individual is not precise enough to be biologically significant (Godfrey-Smith, 2013; Booth, 2014; Chiu and Eberl, 2016; Queller and Strassmann, 2016; Skillings, 2016). Secondly, the claims, assumptions, and implications concerning the biological individuality of holobionts does not seem to be completely supported by our current empirical evidence (Moran and Sloan, 2015; Douglas and Werren, 2016; Hurst, 2017; Bourrat and Griffiths, 2018; Stencel and Wloch-Salamon, 2018). The realization of these difficulties led all these authors to argue that the hypothesis that holobionts are biological individuals is ungrounded, and they should be rather characterized as ecological communities wherein the microorganisms that integrate the host’s microbiota should be taken as environmental factors for the host’s development and functioning. We will call this position *the ecological-community view*.

**FIGURE 1:**
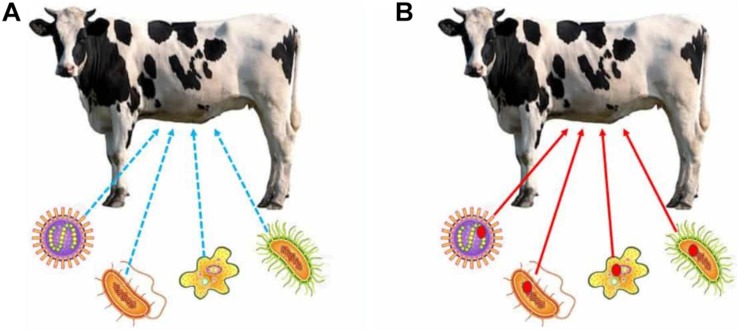
Comparison of the ecological-community view **(A)** and the individual view **(B)**. The blue dashed arrows represent ecological interactions, but not considered from an evolutionary perspective **(A)**, whereas the red arrows represent the evolutionary aspect of these interactions **(B)**. The bacteria stand for hypothetical strains in the cow rumen. In the ecological-community view, herbivory is seen as the result of an ecological interaction, and thus its evolutionary basis does not need to be studied. The individual view, on the contrary, requires studying the evolutionary history of herbivory to unmask the traits of the microbiome that have evolved to make it possible. The red dots in each of the bacterial strains in **(B)** represent traits that have evolved specifically for herbivory, and thus reveal a hologenomic evolutionary history, being most likely fortuitous benefits (rather than etiological adaptations) of the bacterial strains that bear them.

Despite the considerations made by the advocates of the ecological-community view, we suspect that completely disregarding the individual view might be problematic. This is so because the reason why some adaptations have evolved (such as those that are required for dietary, immunological, or reproductive specializations in some animals or plants) would be masked unless the holobiont is taken seriously as *the individual that manifests these adaptations* (Díaz, 2015; Roughgarden et al., 2017; Suárez, 2020). For example, think of the evolution of herbivory in ruminants. In two recent studies, Chiu and Gilbert (2019) and Gilbert (2019) have shown how developmental symbiosis has played a pivotal role in the evolution of this specific dietary lifestyle, to the point that its evolution would have not been possible without the microbial symbionts, insofar as they bear some of the adaptive traits that make herbivory possible. Interestingly, their work is especially revealing, for it shows why an ecological community view would leave herbivory unexplained. Even though we may describe what happens today among ruminants in ecological terms (i.e., conceiving ruminants as an ecological community where some species produce some compounds that others use, the latter transforming these compounds further and making them useful to others, etc.), this level of description would completely mask how herbivory evolved, and why ruminants bear a microbiome that contains certain functional traits rather than others. In other words, the ecological community view would make the evolution of herbivory random, like a fortuitous event of “lucky association” between different species, rather than a causal evolutionary process that depends on natural selection acting on the multispecies community (Figure 1).

Importantly, we are not claiming that the ecological community view cannot (or should not) be applied to understand some of the properties of host–microbiome associations. Our point is rather that relying *exclusively* on the methods that the ecological community approach provides would mask the causal origin of some contemporary host and microbiome traits, whose causal origin would be inappropriately explained. This would be the case since the individual that has historically borne them and thus, the individual on which natural selection has acted so that these traits have historically evolved to become adaptative, would not be recognized (etiological notion of adaptation), being systematically conflated with the individual that happens to bear the trait in its genome now (dispositional notion of adaptation). In other words, some adaptations require the interaction of the host and its microbiota to evolve, while neither the host, nor the taxa that compose the host’s microbiome, would be properly characterized as the individual that manifests them. Therefore, the holobiont must be recognized as the individual that manifests the adaptations that underlie the evolution of some specializations, and hence it is the ultimate reason why these adaptations have evolved and been historically maintained (Mayr, 1961).

## Sanguivory Diet in Vampire Bats

Animals of the order Chiroptera, commonly known as bats, exhibit an important variety of dietary specializations, including specialization to insectivorous, frugivorous, and hematophagous (or sanguivorous) diets. Each of these dietary specializations requires a sophisticated set of morphological, immunological, and physiological adaptations to cope with the challenges posed by the lifestyle that they entail. Furthermore, the order Chiroptera is the only mammal order for which there are three obligate sanguivory species, the three of them belonging to the family commonly known as the “vampire bats” (Phyllostomidae Desmoodontinae). As blood is a challenging dietary source, the fact that that vampire bats are the only mammal family that feeds on it entails that each of the adaptations that make sanguivory diet possible and triggered its evolution must be specific to the Phyllostomidae Desmoodontinae family.

Blood consists mainly of a liquid phase and a dry-matter phase which mainly contains proteins (about 93%), and carbohydrates (about 1%). It provides almost no vitamins, and it could contain high levels of bloodborne pathogens, which are potentially dangerous. Vampire bats have evolved some key adaptations to cope with the challenges posed by this particular lifestyle. These include:

• important morphological changes such as the acquisition of sharp incisor and canine teeth, as well as claw-thumbed wings, that allow them to suck the blood of their prey;
• changes in the sensory apparatus, such as the evolution of sensing capacity to locate the accessible blood vessels in their prey;
• the evolution of adaptations to cope with viscosity, and the possibility of coagulation after ingestion and during digestion;
• the evolution of renal and bladder adaptations to cope with their mainly liquid-based and protein-rich diet (especially related to efficient urea excretion);
• the evolution of adaptations to cope with the risk of iron poisoning;
• the evolution of adaptation to cope with the scarcity of nutrient-availability that is provided in its blood-sucking diet; and
• the evolution of immunological adaptations to cope with bloodborne pathogens, as these are expected to be commonly faced due to their dietary specialization.

All these adaptations, and others, are specific to the Phyllostomidae Desmoodontinae family (as this is the only hematophagous mammal family) and are essential for the evolution of the sanguivory lifestyle of the members of the family. To understand how the sanguivory lifestyle could have evolved, it is important to discover the different adaptations that underlie it, and thus to genetically locate the traits that, by having been naturally selected, have allowed its evolution on the first place.

Mendoza et al. (2018) have recently studied the different nutritional adaptations that underlie the evolution of sanguivory in the common vampire bat, *D. rotundus*. In relation to the sensory adaptations that underlie sanguivory, Mendoza et al. (2018) found that the bat genome had lost its sweet taste genes and had experienced a substantial reduction in the number bitter taste genes. These genes are probably related to homeostasis, and thus their evolution is fundamental for sanguivory. Additionally, they found that the gene *PRKD1*, which modulates the bat’s infrared sensing that allows it to easily locate blood vessels, had undergone positive selection too.

Mendoza et al. (2018) also discovered that *RAB1B* gene and *GJA1* gene had experienced positive selection, probably accounting for gastrointestinal and urinary adaptations to the sanguivory lifestyle, especially coping with the potential challenge of kidney and bladder failure. Regarding the challenge posed by the scarcity of nutrients that are available in a blood-based diet, the common vampire bat has evolved several key adaptations in its genome. These include a positive selection for the gene *REG4*, believed to have a possible effect as an anticoagulant; a positive selection for the genes *PDZD11* and *LAMTOR5*, possibly involved in coping with nutrient scarcity and obtaining an efficient response to nutrient starvation; and a positive selection for *FFAR1* gene, involved in glucose homeostasis, thus hypothetically allowing *D. rotundus* to have an efficient use of the available glucose.

Apart from nutritional adaptations, Mendoza et al. (2018) also found out other traits that were undergoing positive selection in the bat genome. For instance, the antimicrobial gene *RNASE7*, with a possible influence in coping the bloodborne pathogens, showed positive selection. The same was true for the gene *PSMA3*, hypothetically involved in the disposal of excess nitrogen, an expected consequence of a blood-based diet due to its high protein and salt content. Concerning blood coagulation, they found the *PLAT* gene to be undergoing positive selection too, and they further found out that both light and heavy chains of ferritin (an iron-storing protein) were under gene family expansion. Ferritin is important to avoid an excess of iron in the blood flow, which might be triggered due to the high content of iron in blood, which is the only nutritional source of vampire bats.

However, the *genomic adaptations* just described do not seem to be enough to cope with all the challenges posed by sanguivory and, even if they could trigger the evolution of some hematophagous behaviors, they do not seem enough to explain the evolution of *obligatory* sanguivory. Covering that gap, together with these key genomic adaptations, Mendoza et al. (2018) also found that several traits in the common bat’s functional core microbiome were undergoing positive selection to cope with the nutritional challenges posed by sanguivory. For instance, they found an enrichment in some microbial genes in the functional core microbiome, including the microbial gene L-asparaginase, possibly involved in anticoagulation, one of the main challenges of hematophagy. Furthermore, they found an enrichment, and possibly a positive selection for genes involved to carbohydrate metabolism and energy production, including enzymes related to the reverse Krebs cycle, and to the biosynthesis of cofactors and vitamins, such as carotenoid and butyrate. All these genes are speculated to play a key role in coping with the low nutrient availability in the common vampire bat blood-sucking diet. Concerning fat storage and the synthesis of triacylglycerol, Mendoza et al. (2018) found an enrichment in the microbial gene glycerol kinase.

The contribution of the microbiome to sanguivory is not exhausted by nutrition, though. The vampire bat microbiome was discovered to contain a large abundance of protective, antiviral producing bacteria, in comparison to the microbiome of other bats, which suggests that the microbiome might contribute to immunity. Additionally, they also found an enrichment in ferritin, which suggested that the microbiome also collaborated to coping with the excess of iron in the vampire diet. Finally, they found that the microbial gene *ureA*, involved in urea degradation and thus essential to keep the normal functioning of the kidneys, showed an enrichment in the common vampire bat microbiome in relation to other bat species.

### Who Manifests the Adaptations for Sanguivory?

The evolution of sanguivory in the vampire bat triggers the following question. If the evidence gathered by Mendoza et al. (2018) is correct, and the microbiome contributes to sanguivory almost as much as the host does, which entity is the manifestor of adaptation? Or, connecting with what we argued in “Biological Individuals as Manifestors of Adaptation,” if adaptations (in the etiological sense) are only adaptations of an individual in a lineage, which is the individual that bears the adaptations that make sanguivory evolve by cumulative selection? From Mendoza et al.’s (2018) research, it follows that part of these adaptations are genomic adaptations, i.e., adaptations of *D. rotundus*, such as the positive selection of the *GJA1* gene, or the *PSMA3* gene, which allow to cope with some of the challenges of sanguivory.

However, all the changes that are positively selected in the common bat’s genome *alone* fail to explain its hematophagous mode of life, since they only allow bats to cope with some of the challenges posed by sanguivory, but they cannot account for all of the challenges that this type of diet generates. This creates an important explanatory gap: if vampire bats have an obligate blood-sucking diet, but their genome lacks the genetic components that would allow them to cope with all the challenges posed by sanguivory, how is it possible that vampire bats are, in fact, hematophagous? The answer, in Mendoza et al.’s (2018) research, lies in the substantial contributions that the microbial components of the bat microbiome make to sanguivory, including the synthesis of some of the enzymes that avoid blood coagulation, the synthesis of proteins that allow vampire bats to survive despite the scarcity of nutrients in their diet, etc.

Interestingly, Mendoza et al.’s (2018) research also makes another fundamental contribution for understanding the evolution of sanguivory in Chiroptera. In their research, they found that the microbial taxonomic composition in the vampire bat’s microbiome reflected the bat’s phylogenetic influence, with more similarity to the microbiome of insectivorous and carnivorous bats than to frugivorous bats (a pattern known as *phylosymbiosis*, Brooks et al., 2016). However, at the functional level, they found out that the vampire bat’s microbiome was strikingly different to any other bat it was compared to (frugivorous, carnivorous, insectivorous), which according to the authors suggested that the common vampire bat’s microbiome might harbor a specific set of functions highly specialized to its extreme diet.

This observation is important because, as we will argue, it suggests that some of the etiological adaptations that have evolved in the vampire microbiome were not adaptations for any of the bacterial lineages that compose it, but for the host-microbiome system. For now, it is enough to realize that neither the host alone, nor the microbiome alone are the manifestors of the traits that underlie the evolution of sanguivory. Sanguivory evolves as a consequence of the *interaction* between the host and the microbiome and, thus, it seems to be a characteristic of the system formed by both.

## Holobionts as Emergent Individuals

In this section we interpret Mendoza et al.’s (2018) results from the perspective that the entity that manifests the adaptations in the case of bat sanguivory is the holobiont; our argument makes use of the philosophical notion of *emergence*. We divide this section into three parts. In the first part (“Clarifying the Metaphysical Framework: the Notion of Emergence”), we introduce the metaphysical notion of *emergence* and the features attributed to the so-called “emergent-properties.” Specifically, we will clarify the notion of emergence that we are going to use (see also Suárez and Triviño, 2019). In the second part (“The Holobiont as an Emergent Individual That Manifests Etiological Adaptations”), we will illustrate how this way of metaphysically approaching the holobiont offers an accurate framework to explain sanguivory in vampire bats, and in the third (“Biological Consequences of the Emergentist Account of the Holobiont”) we extend the framework to other case studies that appear in the biological literature. Our goal is to show how our emergentist account illuminates some of the empirical results and provides a coherent framework to think about the concept of hologenomic adaptation.

### Clarifying the Metaphysical Framework: The Notion of Emergence

The metaphysical notion of emergence has been widely used among philosophers of biology to characterize some biological properties, such as the features of biochemical networks (Boogerd et al., 2005), the amount of nectar stored in a hive (Mitchell, 2012), or fitness (Triviño and Nuño de la Rosa, 2016).

Emergent properties, in the ontological sense^5^, have two main characteristics: *dependence* and *autonomy* (Sartenaer, 2013). Regarding *dependence*, emergent properties are “higher-level properties” of a system that depend on the “lower-level properties” of the parts that compose that system. Metaphysicians have widely worked on clarifying the kind of dependence that is given between the emergent and the “lower-level properties” (O’Connor, 1994; Humphreys, 1997; Kim, 1999; O’Connor and Wong, 2005). Recently, Jessica Wilson has made a review of the different forms of dependence and has distinguished five types: material composition, fusion, modal covariation, nor-reductive realization and causation (Wilson, 2016). Emergent properties of different systems, then, might depend on their “lower-level properties” in different ways, and no particular form of dependence can be singled out as a necessary one.^6^

Concerning the biological field, we have elsewhere argued that the kind of dependence occurring between some emergent properties that characterize an organism, such as fitness (see Triviño and Nuño de la Rosa, 2016; Triviño, 2019), and its “lower-level properties” is *causal-functional interaction* (Suárez and Triviño, 2019).^7^ In this sense, the emergent property arises as a consequence of the complex causal interactions among the “lower-level parts” of the system. In other words, the “lower-level properties” of the parts of the system *cause* the emergent property to appear (O’Connor and Wong, 2005, p. 664).

Regarding *autonomy*, emergent properties need to introduce a new causal power into the world (O’Connor, 1994; Kim, 1999, 2006). The notion of causal power is, notwithstanding, problematic, as it can be conceived in different ways depending on one’s ontological commitments about properties. Here, we will follow Wilson’s characterization of causal power according to which having a causal power means that the bearer of the property has the capacity to behave in a certain way *given the appropriate circumstances* (Wilson, 2002, 2013, 2016).

The causal power of emergent properties is said to be both autonomous and downwardly exerted. It is autonomous insofar as it is *qualitatively different* from the causal power possessed by the “lower-level properties” that constitute the system. It is downwardly exerted since the system, due to its higher-level properties, is able to exert top–down causation on the “lower level parts” that compose it (O’Connor, 1994, pp. 97–98). This form of causation is conventionally considered as a hallmark of emergence.^8^ In philosophy of biology, it is suggested that the “lower-level parts” of a system behave in ways that they would not behave if the emergent property would not exist due to the constraints created by the higher-level organization that they constitute (Campbell, 1974; Arnellos and Moreno, 2012; Moreno and Mossio, 2015; Green, 2019).

Dependence and autonomy, therefore, are the characteristics that higher-level, i.e., systemic properties need to satisfy to be characterized as “emergent.” In a recent paper, we use this metaphysical framework to argue that holobionts are emergent individuals, insofar as they possess emergent properties (Suárez and Triviño, 2019).^9^ In particular, we argued that holobionts can determine part of the genetic properties of their microbiome. Here, we apply this metaphysical framework of holobionts to provide an interpretation of the results obtained by Mendoza et al. in their study of the evolution of sanguivory in vampire bats. Our aim is to show the usefulness of this metaphysical framework in biological research and how it can shed light on the nature of the holobiont in a way that could be extended to the evolution of other complex specializations in different animal and plant orders.

### The Holobiont as an Emergent Individual That Manifests Etiological Adaptations

In the case studied by Mendoza et al. (2018), the vampire bat holobiont is the individual that realizes sanguivory, and thus the individual that manifests the lifestyle, as we argued in “Who Manifests the Adaptations for Sanguivory?”. In this sense, the vampire bat holobiont, but not the vampire bat host or the vampire bat microbiome, is the entity that bears the adaptive traits that allowed the evolution of sanguivory in the family Phyllostomidae Desmoodontinae. Or, using the two different conceptions of “adaptation” that we had introduced before: all the traits that Mendoza et al. (2018) have proven to show a history of positive selection for the challenges posed by sanguivory on the bat genome, and on its microbiome, are etiological adaptations of the holobiont, rather than of the bacterial taxa that compose the host’s microbiome.

We consider that these results can be properly explained by using the notion of emergence. In this sense, the sanguivory diet can be characterized as an emergent property of a system, i.e., the holobiont. This is so because sanguivory meets the features of *dependence* and *autonomy* that characterize emergent properties.

Regarding *dependence*, sanguivory diet is a property that is not given at the “lower-level parts” that compose the holobiont. Specialization to sanguivory, as well as the traits that evolve to make this specialization possible, only exist as a consequence of the *functional interaction* between the vampire bat and its microbiome. That sanguivory is not a property of the vampire bat or the microbiome but of the holobiont vampire bat-microbiome can be explained from Mendonza et al.’s (2018) results. First, the traits that Mendoza et al. (2018) have shown to be experiencing (or have experienced) positive selection in the microbiome of vampire bats are linked to the specific challenges posed by sanguivory, but not by the challenges posed by every possible lifestyle of the microbial taxa that bear them. This was proven in Mendoza et al.’s (2018) comparison of the taxonomic and functional gut microbiome profiles across different bat species. While gut microbiome variation was scarce at the taxonomic level among bat species, it was strikingly high at the functional level. This suggests that the taxa that compose the bat microbiome will only acquire these traits when they are hosted by vampire bats, but not otherwise. This shows that the traits that underlie sanguivory, and that show a history of positive selection, exist and are transgenerationally maintained through *functional interaction* between the host vampire bat and its microbiome. Without this specific type of interaction, the traits are simply not present, as Mendoza et al.’s (2018) functional analysis of the different types of gut microbiomes suggests. Therefore, as in other cases of emergent properties, sanguivory and, specifically, the traits that make it possible in vampire bats, only exist as a consequence of the interaction between the vampire bat host and its microbiome.

Second, an important consequence of the previous point is that the traits that can be proven to have experienced positive selection in the bat microbiome are not necessarily etiological adaptations for the bacterial taxa that bear them. These traits only appear and become dominant in the bacterial population when the bacterial species reside within the vampire bat holobiont, but do not appear when the same taxa live in symbiosis with other bat hosts, including frugivorous, and insectivorous bats. In this sense, these traits constitute engineering (dispositional) adaptations of the bacterial lineages that have not really been *selected for* their lineages. That is to say, they are the product of natural selection acting on the holobiont, which is the entity that manifests the emergent property of sanguivory.

Concerning *autonomy*, we argue that sanguivory is a *new* property that is not given at “lower-level parts.” As we have shown, this property is not present in vampire bats hosts nor in the microbiome, but it is a property that depends on the causal interaction that occurs between the two of them and is possessed by a higher-level system that we refer to as the holobiont. Insofar as it is a new property, it introduces a new causal power into the world, namely: it allows its bearer, i.e., the holobiont, to behave in a certain way. In this case, to have “sanguivory diet” means that the holobiont vampire bat-microbiome can ingest blood, digest it, and obtain nutrients from it. This is something that neither the vampire bat host, nor the microbiome can do if they are taken separately (see “Who Manifests the Adaptations for Sanguivory?”).

To introduce a new causal power into the world is not sufficient for a property to be emergent, though. As we said in “Clarifying the Metaphysical Framework: the Notion of Emergence,” it is also necessary that its effects are downwardly manifested. The higher-level property, therefore, must allow its bearer to exert top–down causation, that is, to exert causal influence over the parts that compose it. We consider that this feature is also given in the case of sanguivory. In fact, we consider that it is precisely the existence of sanguivory that allows the holobiont vampire bat (insofar as it is the bearer of the property) to exercise a causal power over the “lower-level parts” that compose it (host genome, bacterial lineages), in a way such that some of the traits will be historically maintained for several generations. Importantly, notice that we claim that the emergent character of the property (sanguivory) determines the existence of top-down effects not only on the microbiome traits, but also on the host genome. The evolution of sanguivory, and its maintenance in *D. rotundus*, depends on the evolution of certain traits, and these can evolve both in the bat’s genome, or in the (meta)genome of its microbiome. The traits that have evolved in the microbiome to make sanguivory feasible determine the traits that *have not* evolved in the bat genome, and vice versa, generating thus a reciprocal dynamic that affects to a big extent host genome evolution. In this sense, it does not make sense to argue that the traits that have evolved (or have not evolved) in the host genome to facilitate sanguivory are etiological adaptations of the host: as it happens with the microbiome, attributing the evolution of these traits exclusively to the host would mask their real causal history. The traits that have been acquired/retained by the host genome are hologenomic traits, for they only exist because the holobiont, the entity that realizes sanguivory, exists.^10^

**FIGURE 2:**
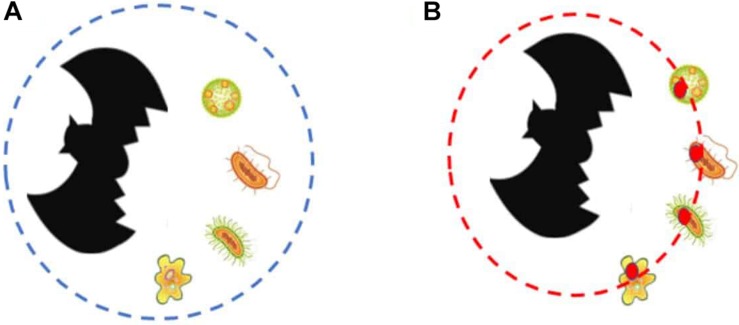
The figures represent a host (bat) plus the set of microbial taxa it interacts with. **(A)** Represents each taxa, and assumes that the individuality of the holobiont consists in the collection of organisms, including the host and the bacterial taxa that reside on its microbiome (represented by a dashed blue circle). **(B)** Represents our emergentist account, according to which the holobiont is the entity composed by the host plus the etiological adaptations that allowed the evolution of sanguivory that are borne by the taxa that compose its microbiome (the adaptive traits are represented by the red circles, and the boundaries of the emergent holobiont are represented by the dashed red circle). These adaptive traits that belong to the emergent holobiont (despite being borne by the bacterial taxa) include the set of genetic components that Mendoza et al. (2018) proved to have been selected to cope with the challenges of sanguivory.

A consequence of this is that the bat holobiont *is the manifestor of adaptation* and therefore, *the individual where the traits that underlie sanguivory in vampire bats have evolved*. And, importantly, the bat holobiont is the entity whose existence causes the microbiome to bear the functional traits that it bears (and that are reflected in the functional analysis) (Figure 2), as well as the evolution of the host genome (those traits that evolve in the microbiome do not evolve in the host genome, and vice versa). This constitutes a case of top–down causation, where the entities at the lower-level acquire part of their properties as a consequence of the effect caused by the property of an entity at the higher level. The reason why these traits exist is thus twofold: on the one hand, they exist now on the bacterial lineages because they are the ones that make the bacteria fit better the environment where they live (dispositional account of adaptation); on the other hand, they have existed historically because they allow the vampire bat holobiont to realize sanguivory, and thus to have the specific lifestyle that it has (etiological account of adaptation). Our point is thus that the traits underlying sanguivory are, in most cases, etiological adaptations of the holobiont, and engineering adaptations of the taxa that compose the microbiome and/or the host genome.^11^

We have already shown that sanguivory can be characterized as an emergent property insofar as it meets the features of dependence and autonomy. In this regard, the holobiont, that is, the system that bears this property, can also be characterized as an emergent entity in a derivational sense since, according to authors such as Bedau (1997), those systems that possess emergent properties can be considered emergent systems. A consequence of our interpretation is that the holobiont is more than a mere epiphenomenal association between hosts and their microbiome: holobionts are emergent entities, insofar as they are the bearers of emergent properties.

In this regard, it is important to clarify that, in characterizing the holobiont as an emergent entity we are not trying to answer any empirical question about any specific biological system. That is, we are not explaining at what point in history sanguivory appears and, therefore, the bat holobiont (as an emergent entity) started to exist. Nor are we studying when, during the ontogeny of the vampire bat host, the host-microbiome association becomes an emergent entity (the holobiont) rather than an aggregate. These are empirical questions that, despite their importance, are besides the scope of this paper for two reasons. First, because they will be different for every biological system (vampire bats, cows, or *D. melanogaster* will have evolved into holobionts differently). And second, because their ontogenetic origin also depends strongly on the system one is concerned with, as well as with the type of behavior that one is trying to explain (sanguivory, herbivory, niche adaptations, etc.). Here, instead, we use metaphysical concepts to explain an already existent biological phenomenon, namely, sanguivory. In addition to that, we are justifying why characterizing the vampire bat-microbiome holobiont as an emergent entity that bears an emergent property is more useful than other alternative accounts of the holobiont for explaining some empirical results, and also to foster new research.^12^

### Biological Consequences of the Emergentist Account of the Holobiont

The emergentist account of the holobiont can be generalized to every other animal or plant, due to the universality of host-microbiome interactions. Thus, we suggest that our framework is accurate to interpret the main biological features of holobionts, and to interpret some of the empirical results that are obtained when a hologenome framework is applied in scientific research. For example, as we advanced in “Biological Individuals as Manifestors of Adaptation,” the evolution of herbivory in ruminants can be explained by assuming that the cow-holobiont is an emergent biological individual (something that Chiu and Gilbert, 2019 explicitly acknowledge). On the one hand, herbivory satisfies the requirement of dependency -for, as Gilbert (2019) argues, it only appears as a consequence of the host-microbiome interaction. On the other, herbivory is an autonomous property, as its existence leads to downward effects on the genome and the microbiome of cows that, we argue, must be manifested in the type of functional traits that have evolved, and the type of dynamics that these traits have followed. Particularly, we hypothesize that origins of herbivory, as a property of the cow-holobiont, leads to the evolution of highly motile traits, many of which will be involved in a high degree of horizontal gene transfer among the microbiota that composes the cow’s rumen.

Another case that can be reinterpreted under our framework is the appearance of hybrid lethality in *Nasonia* wasps (Brucker and Bordenstein, 2013b). In their study, Brucker and Bordenstein proved that hybrid lethality could be “cured” among closely related species of *Nasonia* if their microbiomes were removed. This suggested that hybrid lethality did not result from a genomic incompatibility among related *Nasonia* species, but from a hologenomic incompatibility (cf. Chandler and Turelli, 2014). Brucker and Bordenstein explained their results by appealing to the genetic incompatibilities among the beneficial bacterial communities in related *Nasonia* species. While this interpretation is plausible, we believe that our “emergentist” framework provides a better analysis of the results. In our view, Brucker and Bordenstein clearly proved that hybrid lethality is both a dependent and an autonomous property. It is dependent for it only appears as a consequence of the interaction between the zygotically derived *Nasonia* cells, and the bacteria that compose their microbiome. It is autonomous because once the emergent hologenome that manifests lethality has appeared, the evolution of the genomes of the different (and incompatible) *Nasonia* species and the evolution of their microbiome will follow distinct evolutionary pathways that result, precisely, from the biological possibilities that hybrid incompatibility generates (see also Suárez, 2019, pp. 51–71, for an extensive discussion).^13^

Finally, we also believe that Rosenberg and Zilber-Rosenberg’s study on the evolution of corals -which inspired the hologenome concept of evolution (Rosenberg et al., 2007; Zilber-Rosenberg and Rosenberg, 2008; Rosenberg and Zilber-Rosenberg, 2013)- can be reinterpreted by appealing to the notion that the holobiont is an emergent biological individual. In the late 90s, using the Koch postulates, *Vibrio shiloi* had been deemed responsible for the disease affecting *Oculina patagonica* (Kushmaro et al., 1997). However, some analyses made a few years later showed that *V. shiloi* had disappeared from most of the corals, suggesting that corals had overcome the infection. Reshef et al. (2006) suggested that corals could overcome the infection because their microbiome was rearranged in a way that caused the disappearance of *V. shiloi*. Generalizing from this observation, Rosenberg et al. (2007) and Zilber-Rosenberg and Rosenberg (2008) proposed the hologenome concept of evolution, according to which every animal and plant should be considered an evolving holobiont, together with its microbiome. A key element of their proposal is that the collection of genomes that composed corals evolved as a single unit (thus the choice of the name “*holo*-genome”). However, further evidence disconfirmed Rosenberg and Zilber-Rosenberg’s interpretation of corals’ evolution by showing that there was no transgenerational phylogenetic stability in the microbiome of corals (Hester et al., 2016). We hypothesize that this evidence can be interpreted according to our framework, to argue that some of the traits that caused the disappearance of *V. shiloi* are located on the microbiome, in such a way that they are etiological adaptations of the holobiont, and not of the bacterial taxa that compose it. According to our interpretation, the immunology of corals is an emergent property of the holobiont that is both dependent on host–microbiome interactions, and autonomous in the sense of causing downward effects that alter the evolution of both the genome of corals, and of its microbiome. A consequence of this view is that, as Hester et al. (2016) observed, transgenerational stability at the level of the species that compose the microbiome of corals is not to be expected while, as Rosenberg and Zilber-Rosenberg argued, the emergent effect that causes the disappearance of *V. shiloi* is expected to remain.

Now we have explained what the emergentist account of the holobiont entails and how it could be applied to illuminate some aspects of contemporary biological research, a new question arises. Contemporary research on the microbiome has suggested that the species that compose the microbiome of a host may suffer dramatic changes during its lifetime, some of which may even lead to a full replacement of the species that compose the microbiome (Gilbert and Chiu, 2015). Grounded on this, some researchers have denied the “individuality” status to holobionts, as they lack stable properties underlying their temporal identity, which renders the holobiont as a “fluid” entity that is constantly changing and becoming a different individual. Although we believe this is a fair criticism to the notion that the holobiont is the individual composed by a host and the totality of *taxa or species* that compose its microbiome, we do not think that it can be applied to the emergentist conception of the holobiont we advocate here. To explain why, in the next section we provide an account of the identity of the holobiont that builds on its emergent nature, and that appeals to the notion of inter-identity.

## The Inter-Identity of the Holobiont

The question about the identity of a biological individual concerns the conditions that make a biological individual the same entity despite the continual changes^14^ it experiences through time (Bouchard, 2013).^15^ In the case of holobionts, the question of identity can be expressed as follows: How can we determine that a holobiont is one and the same through a period of time, *t*_1_-*t*_10_, given that its properties change through this period of time? In fact, holobionts may experience changes in both its component parts and its qualitative features between *t*_1_ and *t*_10_. Thus, how can we know whether a holobiont at *t*_1_ is the same holobiont at *t*_10_? Is the identity of the holobiont different from the identity of the host, or does each holobiont live as long as each host lives? How would a change in the microbiome of a host affect the identity of the holobiont? As, in our account, the holobiont is the entity that emerges from the interaction between the parts that compose it -i.e., the host and its microbiome- it becomes necessary to specify the type of changes that it could experience while being the same individual.

In the most recent literature, the holobiont is conceived of as the individual composed by a host plus the *species or taxa* that compose its microbiome. Thus, in that view, most authors had argued that the identity of the holobiont is not temporally preserved, as the microbiome species composition can sometimes be very unstable during the lifetime of the host.^16^ Chiu and Eberl (2016) have recently presented the most elaborated version of this argument. They join three pieces of evidence to support their view. First, the bacterial species of the microbiome that interact with a host are usually the result of a process of ecological filtering, rather than the result of a process of host filtering (Moran and Sloan, 2015; Douglas and Werren, 2016; Mazel et al., 2018). In their view, only the latter would suggest coevolution and thus, individuality, but not the former. Second, the microbiome is largely interchangeable during the lifespan of a host. A host can interact with different species of microorganisms during its life, and the species composition of its microbiome is fluctuant (Gilbert and Chiu, 2015). Third, the species that compose the microbiome of a host are shared among many different hosts at different times, and thus, the microbiome is not a proper part of the host, which implies that the holobiont is not a biological individual.

Even though Chiu and Eberl’s arguments pose a serious challenge to the individuality of the holobiont, and hence, to its identity, we do not believe they are correct, as we have extensively argued somewhere else (Triviño, 2019, pp. 198–233). Firstly, from a biological perspective, the individuality of the emergent holobiont results from the shared history of the adaptive traits carried by the microorganisms of the microbiome and interacting with the host genome. This contrasts sharply with the idea, which we consider incorrect, according to which the individuality of the holobiont results from the interaction between the host genome and the different taxa or species that compose it. Secondly, from a metaphysical point of view, contemporary metaphysical theories of persistence explain that a proper part of an individual can be contingent, interchangeable and shared without necessarily affecting the identity of this individual (McCall and Lowe, 2003, 2006; Miller, 2005, 2010).

Assuming that our position is correct, we still need to explain what the identity conditions of holobionts are according to our emergentist view. Due to their “interactive” nature, we propose that the identity of holobionts is a form of *inter-identity*, that is, a kind of identity that depends on the maintenance of the interaction between the host and the adaptive, etiological traits that are borne by its microbiome and thus, on the persistence conditions of both of them.

The persistence conditions of an entity refer to those changes that the entity can support without ceasing to exist, that is, without losing its identity (Lowe, 2002). The persistence conditions vary depending on the nature of the entity one is considering. For instance, the persistence conditions of a watch include the possibility of disassembling the watch into its mechanical components, and reassembling it, without the watch ceasing to exist.^17^ Conversely, an organism cannot normally persist if it is decomposed into parts because, as some people have argued, the nature of organisms is such that their persistence conditions are radically different from those that make machines possible (Nicholson, 2013, 2019). Of course, this does not mean that organisms do not tolerate any kind of replacements in their parts without losing their identity. For instance, an organism can lose parts of their body without ceasing to exist, or they can have their organs replaced by other organs (e.g., transplants). However, they cannot, in most cases, tolerate being completely decomposed into parts and reassembled (although there are some exceptions, e.g., some plathelminths).

Concerning holobionts, in order to explain how a holobiont at *t*_1_ is the same individual at *t*_10_, for instance, we need to take into account its persistence conditions. In this regard, our emergentist view on holobiont individuality entails that the persistence conditions of holobionts include some changes in their constituent parts, namely the host and its microbiome. Changes in the constituent parts that are tolerated include processes such as cell turnover in the host, or “bacterial turnover” in the microbiome, and changes in the species that compose the microbiome, provided these replacements do not affect the adaptive traits that define the boundaries of the holobiont as an emergent individual that manifests these adaptations. Additionally, the emergentist account of the holobiont also tolerates some changes that affect the qualitative properties that characterize each of these parts, including changes in the relative abundances of the species of the microbiome (e.g., changes in their densities), or changes in some organs of the host (such as some organ loses, or some organ replacements).^18^

Drawing upon the example of the vampire bat holobiont, we claim that, *with regard to the host*, it is possible that the bat-host loses some of its parts during its lifetime, such as some hairs, or some of its teeth, such that it is possible for the bat that composes the holobiont to have twenty teeth at *t*_1_, whereas it only has nineteen at *t*_5_. The possible range of changes we are referring to includes both changes in the *components* and changes on the *qualities* of the bat. And these are changes that the bat, due to its nature, can support without ceasing to exist, and therefore, without losing its identity. How is the identity of the bat related to the identity of the holobiont? We claim that the vampire bat holobiont does not lose its identity as a consequence of any change in the host that does not affect the identity of the latter. This is so because the identity of the bat-holobiont is a result of the interaction between the bat and its microbiome, and any change in the bat that does not alter its persistence conditions does not affect the identity of the holobiont. In other words, insofar as the bat is a component of the holobiont, it is possible to claim that, at *t*_1_, the bat-holobiont has twenty teeth whereas at *t*_5_ the bat-holobiont is still the same, but it only has nineteen teeth. Since this kind of change is part of the persistence conditions of the holobiont, it continues being the same one despite the changes it experienced. Losing some hairs or losing a few teeth may definitely affect the fitness of the bat and, indirectly, the fitness of the bat-holobiont. However, it does not affect its capacity to interact with its microbiome, and thus the vampire bat-holobiont does not cease to exist.

*The case of the microbiome* is, however, more complex, for the microbiome can be reassembled and it can possibly even be completely replaced during the life of the holobiont. The question is, thus, what kind of changes in the microbiome would not affect the persistence conditions of the holobiont. In this regard, we consider that a change in the species composition of the microbiome is a change that the holobiont can support, and therefore, it is part of its persistence conditions, such that the holobiont at *t*_1_ is the same one as the holobiont at *t*_5_ regardless of the bacterial species that compose the microbiome that interacts with the host (Triviño, 2019, pp. 198–233). Following the ideas of some biologists (Burke et al., 2011; Taxis et al., 2015; Catania et al., 2016; Louca et al., 2016; Doolittle and Booth, 2017; Lemanceau et al., 2017), we consider the nature of the holobiont to be such that it can support changes in its microbiome as long as *the functions* that the microbiome performs for the host *are maintained*.

We can use one example to illustrate this. In a study on the aphid-*Buchnera* symbiosis, Koga et al. (2003) successfully replaced the *Buchnera aphidicola* in a group of aphids by a different symbiont (pea aphid secondary symbiont), despite the obligate nature of the aphid-*B. aphidicola* association. And, in a recent study, Chong and Moran (2018) have shown how the aphids from the *Geopemphigus* species have naturally replaced their *B. aphidicola* for a different symbiont closely related to the phylum Bacteroides. These types of species replacements can be tolerated without the holobiont losing its identity insofar as the functions that the microbiota realizes are the same, which strongly suggests that the adaptive traits that are arguably etiological adaptations of the holobiont still remain. In this regard, it is not relevant for the microbiome that interacts with a host to be of a species *S*_1_ or *S*_2_, as long as it properly performs the functions it has to perform in its interaction with the host, i.e., as long as the new species carries the traits that are holobiont adaptations. Thus, the holobiont can support structural changes in its microbiota species-composition without losing its identity.

In the case considered here of the evolution of sanguivory in vampire bats, the function of the microbiome depends on its capacity to guarantee that its interaction with the bat-host allows the bat-holobiont to realize sanguivory. Appealing to the notion of function is important since, as we argued, not every bacterial species in the microbiome is able to perform the accurate function when interacting with a host, either because they lack the genes/traits, or the capacity to carry out the required activities (Maynard-Smith and Szathmary, 1995; Dethlefsen et al., 2007). Thus, not all the taxa that compose the microbiome are parts whose elimination would alter the persistence conditions of the holobiont. Only these elements that perform an accurate function (i.e., only those traits that, according to our account, are etiological adaptations of the holobiont) are taken as parts of the holobiont whose disappearance would lead to a disruption in the identity conditions of the holobiont. Given this, as long as these traits and the function they perform is maintained, the rest of the elements that compose the microbiome of a holobiont can change without affecting its persistence, and therefore, without affecting its identity. Thus, those changes in the microbiome that do not affect its function (and thus, its functional traits) are possible without affecting the persistence conditions of the holobiont. In this vein, the identity of the holobiont is a result of the interactions between the host and the etiological adaptations of the microbiome that are etiological adaptations of the holobiont. This form of identity, insofar as it occurs among different individuals (i.e., the traits are borne by different genomes), takes the form of an *inter-identity*.

So far, we have explained the persistence conditions of holobionts, i.e., the type of changes that holobionts could support without losing their identity. This, though, raises a question about the type of changes that would directly affect holobiont persistence and, thus, holobiont identity. According to our emergentist approach to the individuality of holobionts, there are three different -although related- changes the holobiont cannot support: the total absence of a host, the total absence of the microbiome, or the absence of an adequately mediated host-microbiome interaction. This is so since, without any of these relata, the set of biological and dynamical processes that gives rise to the holobiont, and to its specific properties, disappears. If at some point between *t*_1_ and *t*_10_, the host that interacts with the microbiome and that is a part of the holobiont is killed, the holobiont could not continue persisting and therefore, its identity would be lost. In the same way, if, at some point between *t*_1_ and *t*_10_, the holobiont loses its microbiome, then it would disappear as well. In the former case, there would only be a set of microbes living on a dead body, but not a holobiont, whereas in the second there will only be a bat, but this would not be able to manifest sanguivory, as it would lack all the microbiome traits that make it possible.^19^ Finally, a third possibility that holobionts would not support is the lack of host-microbiome *interaction*. If at some point in time the host and the microbiome are together, but they stop interacting -either because the host dies, or because the microbiome becomes “denaturalized,” i.e., it loses its functional specificity-, the holobiont loses its persistence conditions and thus it ceases existing. This is so insofar as the holobiont results from the interaction between the host and its microbiome, which generates etiological adaptations that either of them would lack separately.

Taking all of this into account, we conclude that the identity of the holobiont is established both by the properties of its component parts that give rise to the existence of the holobiont as an emergent individual, plus those that result from their interactions with each other. Therefore, we consider that the identity of the holobiont can be better conceived of as a form of *inter-identity*. That is, an identity that results from the interaction of different elements whose identities contribute, simultaneously, to the identity of the emergent individual (the holobiont). The way in which the holobiont maintains its identity, thus, is through maintaining its etiological adaptations, which are the ones whose evolutionary existence is explained because the holobiont exists.

## Conclusion

In this paper, we have shown the utility of approaching pressing biological questions by appealing to metaphysical notions. Our approach follows a growing tendency in contemporary biological and philosophical research that consists in combining scientific practice with the use of the metaphysical discourse to clarify some scientific debates (Boogerd et al., 2005; Dupré, 2012, 2015; Mumford and Tugby, 2013; Guay and Pradeu, 2014; Austin, 2016, 2017; Waters, 2017; Austin and Nuño de la Rosa, 2018; Nicholson and Dupré, 2018; Laplane et al., 2019; Triviño, 2019). Concretely, we have shown how approaching the concept of holobiont adaptation by appealing to the notions of *emergence* and *inter-identity* allows to shed light on some of the perceived issues in contemporary hologenome literature. In this regard, we have shown that the concept of the holobiont is indispensable if one aims to explain the etiological origin of some adaptive traits, because the historical reason why these traits have not become extinct lies in their contribution to allow the existence of a particular phenotype in the holobiont (sanguivory, herbivory, niche adaptations, etc.), rather than in their contribution to the fitness of the bacterial taxa that bear them. For that reason, we argued, these traits are etiological adaptations of the holobiont and dispositional adaptations of the taxa that compose the microbiome. This view of the holobiont as an emergent biological individual that manifests adaptations allows to capture this evolutionary dimension of the holobiont without equating the individuality of the holobiont to the co-speciation of the taxa that compose it^20^.

Secondly, we have developed the concept of inter-identity to account for the persistence conditions of the holobiont. We argued that some criticisms to the individuality of the holobiont are based on the lack of clarity about the persistence conditions of the holobiont. Concretely, they are based on the notion that the disappearance or partial substitution of some of the species or taxa that compose the microbiome of a host would lead to the destruction of the identity of the holobiont. We have built on our emergentist conception of the holobiont to explain why that characterization of the identity conditions of the holobiont is mistaken, and have elaborated the notion that the holobiont can be considered the same entity insofar as the interactions between the host and the etiological adaptations of its microbiome (but not necessarily the taxa that bear them) are maintained.

## Author Contributions

The authors declare they have contributed equally. Both endorse the ideas expressed in the manuscript, and agreed with ITS publication.

## Conflict of Interest

The authors declare that the research was conducted in the absence of any commercial or financial relationships that could be construed as a potential conflict of interest.

## Footnotes

1 We will use “adaptation” and “adaptive trait” as synonyms; that is to say, a trait will be considered an adaptation if and only if it is an adaptive trait.

2 Besides etiological and dispositional approaches, some authors have proposed a pluralist account to biological functions (Millikan, 1989; Godfrey-Smith, 1993, 1994) according to which both approaches need to be considered in order to explain different aspects of biological phenomena. Alternatively, unitary accounts argue for the possibility of elaborating a theoretical framework that covers the advantages of both etiological and dispositional accounts of biological functions (Kitcher, 1993; Walsh, 1996; Mossio et al., 2009). Since it is not the purpose of the paper to go deep in the characterization of the different accounts of functions and adaptations, we just present here the two main accounts of functions that have been offered.

3 Elisabeth Lloyd also uses the concept of “beneficiary of adaptation,” to refer to the entity that *ultimately benefits*, in the long term, of the adapting process. She does not develop the notion further, but for what she says, she seems to be referring to the set of genes or alleles that are the long-term survivors of the selection process. To avoid misunderstandings, in this paper we will not discuss Lloyd’s concept of “beneficiary of adaptation.” Thus, when we say that an entity (e.g., a bacterial taxon) opportunistically *benefits* from having a trait, we do not mean in any sense that the bacterial taxon is the beneficiary of that adaptation, but rather than it obtains an immediate benefit of bearing it in a particular context (the host niche).

4 One reviewer has correctly pointed out that the literature on biological individuality is far larger than we show, and thus our election of the concept of “manifestor of adaptation” to pick up biological individuals seems ungrounded, or de-contextualized. We agree with him/her that the debate about biological individuality is large, and there are many other conceptions that may also deserve discussion -e.g., Darwinian individuality (Godfrey-Smith, 2009, 2015), or immunological individuality (Pradeu, 2010, 2012), as the reviewer correctly points out. However, we do not agree that our choice of the concept of manifestor of adaptation is ungrounded: on the one hand, our paper is about the concept of hologenomic *adaptation*, and thus it seems that the more appropriate concept of biological individuality to discuss adaptation is the concept of *manifestor of adaptation*, which was precisely envisioned for that task. On the other, because the concept of the holobiont has already been discussed extensively from most of the other perspectives (e.g., Suárez, 2018 for a review), while the concept of manifestor of adaptation had only be referred to in some papers (e.g., Roughgarden et al., 2017; Lloyd and Wade, 2019), but not adequately developed.

5 *Ontological emergence* refers to a kind of objective properties that exist in the world, whereas *epistemological emergence* refers to those properties that humans characterize as such due to our own limitations in our way of knowing or explaining them (van Gulick, 2001; Wilson, 2016). Although there is a relation between ontological emergence and epistemological emergence, this relation is something that needs to be studied separately (see Wilson, 2016). As our main interest in this paper is ontological, we will not explore the epistemological consequences that follow from the ontological characterization of holobionts as emergent entities. The important idea here is that we do not identify, as other authors do (see Chalmers, 1996; Bedau, 1997; Silberstein and McGeever, 1999), epistemological emergence with unpredictability, and ontological emergence with irreducibility. Notions such as prediction and reduction are part of the epistemological characterization of emergent properties, and they are not at stake when we refer to ontological emergent properties.

6 For example, the determinable/determinate relation -e.g., the relation between shape and its specifications (rectangular, oval, etc.), or the relation between color (red) and shades of color (maroon)- is said to be given by means of non-reductive realization (Wilson, 2016), whereas the most common form of dependence in the field of quantum mechanics is considered to be fusion (Silberstein and McGeever, 1999).

7 For the characterization of the notion of dependence based on causation see O’Connor and Wong (2005) and Wilson (2016).

8 We will use the concept of “top–down effects” to refer to what philosophers conventionally call “downward causation.”

9 Our account of the individuality of the holobiont as an emergent entity differs from other accounts that have appeared in the biological and philosophical literature. Previous accounts of the individuality of the holobiont have emphasized its interactive nature (Zilber-Rosenberg and Rosenberg, 2008; Dupré and O’Malley, 2009; Gilbert et al., 2012; Rosenberg and Zilber-Rosenberg, 2013; Gilbert and Tauber, 2016; Gilbert, 2017). However, they had not paid enough attention to the capacity of the holobiont to downwardly act on the parts at its lower-level, a capacity that derives from its emergent nature as the manifestor of adaptation. Drawing especially on this second feature, we have argued that the holobiont is an emergent individual due to its capacity to realize sanguivory and, as a consequence, to downwardly act on the “lower-level parts” that compose it. Our account is thus new in the sense that it introduces an explicit view about how downward or top-down causation can be possible in holobionts, and why it is important to consider it.

10 Even though Mendoza et al. (2018) do not specifically study the influence of the microbiome on the host genome (although this can be inferred from their results, and their genomic analysis), a very recent study by Rudman et al. (2019) on *Drosophila melanogaster* clearly reflects this dimension, and thus the top-down effect that holobiont organization can have on the host genome. Concretely, in their study, Rudman et al. proved that the microbiome shapes the pattern and process of genomic adaptation, playing an important role in host genomic evolution. In our view, though, this role of the microbiome is a consequence of the top–down effects that the holobiont has on each of its components.

11 In a sense, as Adrian Stencel (personal communication) has pointed out, our emergentist view of the holobiont sees the hologenome as an “extended genome” (rather than an *holo-*genome, if “*holo*” refers to the set formed by the host genome plus the collection of microbial genomes), if the latter is conceived as a collection of genetic material in a lineage that, due to common interests, tends to favor similar phenotypes (Stencel and Crespi, 2013). Even though we agree with him that this would be possible, we still believe that the connection between both ideas requires further development.

12 We would like to thank the useful comments of an anonymous reviewer who made us notice this point.

13 For a different interpretation of Brucker and Bordenstein’s results, see Stencel and Wloch-Salamon (2018).

14 This claim is metaphysically correct as long as the changes do not affect the own essence of the entity itself. This would occur if the change causes the entity to cease existing, for instance (Lowe, 2002).

15 This aspect of identity refers to the so-called “persistence question.” There is also another form of approaching the identity question, i.e., by considering the conditions that make possible to differentiate one particular entity from another, numerically distinct. This is the so-called “distinguishability question” (Wiggins, 2001; see also Lewontin, 2000; Pradeu and Carosella, 2006). In this paper, we will only focus on the persistence question when talking about the identity of the holobiont as a biological individual. The distinguishability question is easier to solve insofar as two holobionts can be distinguished due to the different host that compose them (Bordenstein and Theis, 2016).

16 This claim needs some clarification, for it may lead to a misunderstanding. Contemporary microbiome research suggests that once the microbiome is acquired, its composition remains more or less stable during the host’s lifetime. However, it may also suffer severe environmentally driven perturbations, or it can also change in different stages of the life of the host (birth, aging, etc.) (Gilbert and Chiu, 2015; Uhr et al., 2019). This is the type of instability we refer to in this section.

17 In this regard, it is important to specify that the watch can be decomposed only into its mechanical components, and this requirement is part of its persistence conditions. It is not possible, for example, to decompose the watch into atoms without it ceasing to exist, for its nature does not allow this possibility to occur (Lowe, 2002).

18 In this case, we are considering changes that are part of the persistence conditions of both, the host and the microbiome.

19 Indeed, some of the experimental procedures that are used to study host–microbiome relationships clearly illustrate the point we are making about the persistence conditions of holobionts. For instance, Brucker and Bordenstein (2013b) experimentally proved that hybrid lethality disappeared if the microbiome is removed. And the same type of reasoning can be applied to vampire bats, or to herbivory.

20 Lloyd and Wade’s (2019) concept of “demibiont” would have similar consequences.
